# Tarsal tunnel syndrome: clinical insights, vascular etiologies, and the role of ultrasonography in the diagnosis

**DOI:** 10.1590/0100-3984.2025.0053

**Published:** 2025-11-07

**Authors:** Ocacir de Souza Reis Soares, Márcio Luís Duarte

**Affiliations:** 1 Clínica Radiológica Ocacir Soares, Presidente Prudente, SP, Brazil.; 2 Universidade de Ribeirão Preto Campus Guarujá, Guarujá, SP, Brazil.; 3 Diagnósticos da América S.A, São Paulo, SP, Brazil.

**Keywords:** Ultrasonography, Tarsal tunnel syndrome, Tibial nerve, Vascular diseases, Ultrassonografia, Síndrome do túnel do tarso, Nervo tibial, Doenças vasculares

## Abstract

Tarsal tunnel syndrome results from compression or damage to the tibial nerve or its branches as they pass through the tarsal tunnel beneath the flexor retinaculum. Diagnosing tarsal tunnel syndrome is challenging because of nonspecific symptoms that overlap with those of other lower limb pathologies. Vascular disorders are common causes but are often overlooked. High-resolution ultrasonography has emerged as a valuable diagnostic tool, offering advantages over other imaging methods. This technique allows real-time assessment, identifying the factors that cause vascular compression and improving diagnostic accuracy. Increased awareness of the vascular contributions to tarsal tunnel syndrome can promote early diagnosis and improve treatment outcomes.

## Abstract (pt)

A síndrome do túnel tarsal ocorre por compressão ou dano ao nervo tibial ou seus ramos ao passarem pelo túnel tarsal, abaixo do retináculo flexor. O diagnóstico é desafiador em razão dos sintomas clínicos inespecíficos, que podem se sobrepor a outras afecções nos membros inferiores. Distúrbios vasculares são causas comuns, mas frequentemente negligenciadas. A ultrassonografia de alta resolução emergiu como uma ferramenta diagnóstica valiosa, oferecendo vantagens sobre outros métodos de imagem. Essa técnica permite a avaliação em tempo real, identificando fatores compressivos vasculares e melhorando a precisão diagnóstica. O aumento da conscientização sobre as causas vasculares pode melhorar os resultados clínicos.

## INTRODUCTION

Tarsal tunnel syndrome (TTS) arises due to damage to the tibial nerve or its branches as they traverse the tarsal tunnel beneath the flexor retinaculum on the medial side of the ankle^(1)^. This condition is diagnosed on the basis of clinical criteria, which are often nonspecific and can mimic various other lower limb diseases. Consequently, TTS is frequently overlooked and underdiagnosed^(2)^. TTS has multiple etiologies, with vascular disorders being among the most common.

The symptoms of TTS vary but commonly include shooting pain, discomfort with prolonged standing or walking, numbness, tingling, or burning sensations in the foot, particularly the distal foot or toes^(3^,^4)^. These symptoms are often exacerbated during physical activity, walking or at rest, particularly at night^(4)^. In addition, dorsiflexion and eversion of the foot may trigger symptoms^(4)^. Physical examination may reveal structural deformities, such as varus or valgus alignment, which can result in nerve traction and associated pain, at rest and during movement^(4)^.

A definitive diagnosis of TTS requires confirmation of focal tibial nerve pathology within the tarsal tunnel, achieved through nerve conduction studies or imaging examinations^(5)^. The diagnosis of TTS is challenging^(5)^. Clinical assessments based solely on sensory abnormalities lack specificity, especially in patients with concurrent polyneuropathy^(5)^. The Tinel test, albeit widely used, is limited by significant interexaminer variability, which reduces its specificity^(5)^.

This pictorial essay aims to provide an educational overview of the vascular causes of TTS, highlighting the practical use of high-resolution ultrasonography.

## ANATOMY OF THE TARSAL TUNNEL

The tarsal tunnel is a narrow area formed by osteoligamentous structures and is covered by a thin flexor retinaculum. The roof of the tunnel is formed by the flexor retinaculum, and the floor is made up of the medial walls of the talus, calcaneus, and distal tibia. The tunnel has a proximal portion, which contains the flexor tendons and the tibial neurovascular bundle, and a distal portion located below the malleolus, between the abductor hallucis muscle and quadratus plantae. Compression of the tibial nerve or its terminal branches can lead to compression neuropathy^(1)^.

On ultrasound, both plantar nerves are visible in the distal tarsal tunnel, with the medial plantar nerve located anteromedially and the lateral plantar nerve located posterolaterally. The medial calcaneal nerve is difficult to visualize on ultrasound but can be identified on magnetic resonance imaging (MRI). The inferior calcaneal nerve (or Baxter’s nerve) arises from the lateral plantar nerve and travels beneath the abductor hallucis muscle^(1)^.

## ULTRASONOGRAPHY: A KEY TOOL IN DIAGNOSING TTS

Ultrasonography offers superior resolution compared to MRI for visualizing the superficial structures of the tarsal tunnel. Given the complex, variable nerve distribution in the foot, ultrasonography has become a vital diagnostic tool for TTS, providing detailed imaging of the tibial nerve and its branches. It is especially useful for identifying the precise site of nerve entrapment and determining its underlying cause^(5)^.

High-resolution ultrasonography has several advantages over MRI, including the following^(4)^:

- Widespread availability
- Lower cost
- Superior spatial resolution
- Faster imaging with the ‘elevator’ axial scanning technique, which involves moving the transducer smoothly along the course of the nerve in a perpendicular plane to create dynamic, continuous cross-sectional views, thus facilitating rapid identification of focal entrapment or vascular compression sites
- Dynamic and comparative assessments
- Capability of obtaining images with the patient in the standing position
- Detection of the Tinel sign through sustained pressure or tapping
- Integration of color/power Doppler imaging, which is particularly beneficial for diagnosing vascular conditions.

### Practical diagnostic approach to TTS with ultrasonography

Various studies have addressed the use of ultrasonography in the diagnosis of TTS^(6^,^7)^. Those studies described a step-by-step practical approach to diagnosing the syndrome, as described below.

*Step 1: Clinical suspicion*

- Take a detailed history: ask about pain, paresthesia, numbness, and a burning sensation.
- Note symptom exacerbation with standing, walking, or at rest (especially at night).

*Step 2: Physical examination*

- Inspect foot posture (e.g., flatfoot and varus/valgus).
- Perform the Tinel sign test over the tarsal tunnel.
- Check for visible or palpable varicosities.

*Step 3: Ultrasonographic examination – supine position*

- Perform axial and longitudinal scans with a high-resolution probe.
- Evaluate tibial nerve and branches for enlargement or displacement.
- Identify compressive causes: masses, varicosities, aneurysms, thrombosis, or kinking of an artery.

*Step 4: Ultrasonographic examination – standing position (weight-bearing)*

- Repeat scan with the patient standing to detect dynamic vascular changes, including increased vein diameter and displacement of structures during load-bearing.
- Use color and power Doppler to assess flow.

*Step 5: Doppler interpretation*

- Check for slow flow, reflux, or turbulence.
- Look for signs of thrombosis (absent flow or intraluminal echoes).

*Step 6: Correlation and differential diagnosis*

- Correlate imaging with symptoms.
- Rule out other causes, such as plantar fasciitis, Baxter’s neuropathy, and peripheral neuropathy.

*Step 7: Report and management*

- Highlight vascular compression (if present).
- Recommend further studies (e.g., contrast-enhanced MRI) if needed for masses or cases with inconclusive findings.
- Suggest an appropriate referral for conservative or surgical management.

### Vascular disorders as causes of TTS

Vascular disorders are among the main causes of TTS. According to a study conducted by Fantino^(4)^, varicose plantar veins in the distal tarsal tunnel constitute the most common vascular cause of TTS.

#### Varicose plantar vein

**Figure 1:**
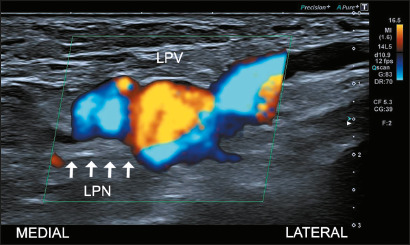
Vein ectasia. Sagittal oblique Doppler ultrasound image of the right ankle, showing a dilated, tortuous lateral plantar vein (LPV) in contact with the lateral plantar nerve (LPN) within the distal tarsal tunnel.

Varicose plantar veins are among the most common vascular causes of TTS. These veins are typically dilated (diameter ≥ 5 mm), tortuous, and associated with signs of venous stasis. Their dilation often becomes more pronounced when the patient is in a standing position^(1^,^7)^, as illustrated in Figure 1.

#### Tibial venous aneurysm

**Figure 2:**
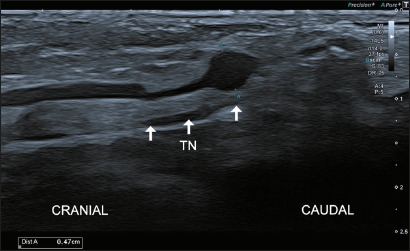
Venous aneurysm. Sagittal ultrasound imaging of the tarsal tunnel on the right ankle demonstrates a focal dilatation of the tibial vein in contact with the tibial nerve (TN).

Tibial venous aneurysms (Figure 2), which lead to vein enlargement, are also recognized contributors to TTS. This condition places the veins in direct contact with the tibial nerve, which may result in nerve compression and related symptoms^(1^,^7)^.

#### Thrombosis

**Figure 3:**
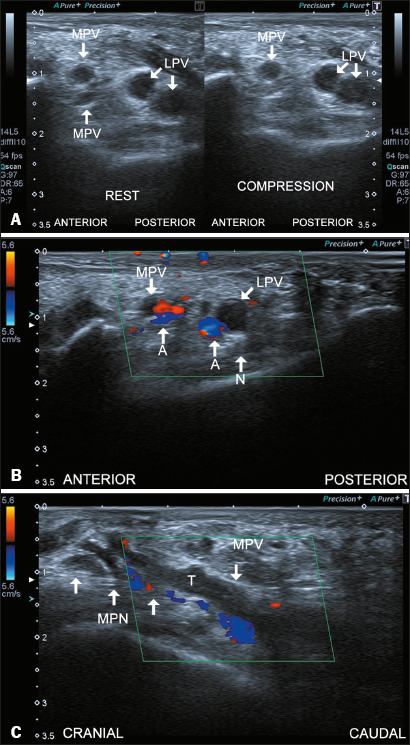
Thrombosis. **A:** Axial ultrasound image of the right ankle at rest and during compression, demonstrating an intraluminal thrombus in a medial plantar vein (MPV) and in both lateral plantar veins (LPVs). Note that one of the MPVs collapses during compression, while the other three thrombus-containing veins do not. **B:** Axial Doppler ultrasound image showing the absence of flow in the MPV and LPV. (A, artery; N, nerve). **C:** Sagittal Doppler ultrasound image demonstrating a thrombus (T) in the dilated MPV, which is in contact with the medial plantar nerve (MPN).

Thrombosis (Figure 3), leading to the expansion of veins, is also a known factor in the development of TTS. Such vascular changes bring the veins into direct contact with the tibial or plantar nerve, potentially causing compression and related symptoms^(1^,^7)^.

#### Kinking of the tibial artery

**Figure 4:**
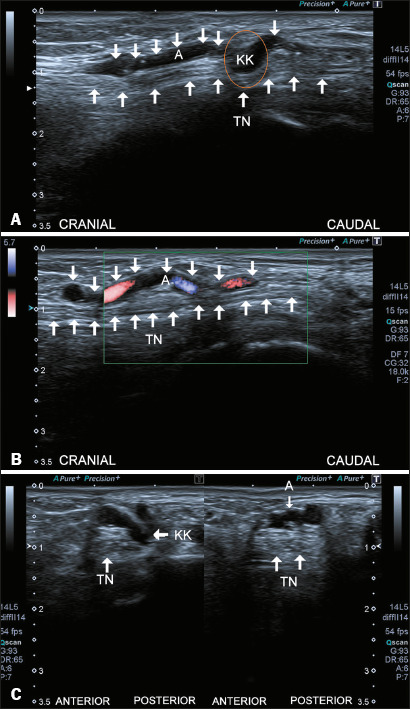
Kinking of the tibial artery. Sagittal ultrasound images of the right ankle without and with color Doppler (**A** and **B**, respectively) demonstrate the kinking of the tibial artery over the tibial nerve (orange circle). **C**: Axial imaging at the level of the kinking reveals that the artery is misaligned with the veins and comes into contact with the tibial nerve (TN, left image), while at a proximal level, normal arterial alignment is observed (right image). It should be borne in mind that tibial artery kinking is not uncommon. However, only cases in which it compresses the tibial nerve may lead to TTS. (KK, kinking; A, artery).

Although kinking of the tibial artery is not uncommon, only cases where the kinked artery directly impinges on or compresses the nerve are clinically relevant to TTS^(1^,^7)^, as shown in Figure 4.

#### Vascular malformations

**Figure 5:**
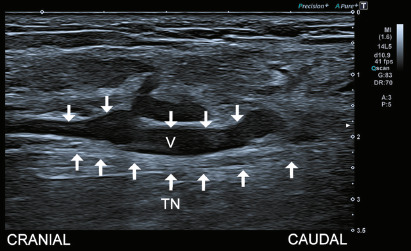
Vascular malformation in a patient with Klippel-Trénaunay syndrome. Sagittal ultrasound image of the right ankle, showing dilated, malformed tibial veins in contact with the tibial nerve (TN). (V, vein).

Venous malformations such as Klippel-Trénaunay syndrome can also impinge on the tibial nerve in the tarsal tunnel, leading to TTS^(8)^, as depicted in Figure 5.

### Importance of accurate imaging

A thorough imaging assessment is essential to differentiate true vascular causes of TTS from incidental findings that do not cause nerve compression, given that false positives can occur^(9)^. High-resolution imaging techniques, combined with a detailed understanding of vascular anatomy, help identify the precise etiology^(4^,^10)^. Key imaging findings for diagnosing TTS^(5)^, including those caused by vascular disorders, are summarized in Table 1. These findings highlight the importance of careful evaluation to increase diagnostic accuracy and inform decisions regarding the appropriate treatment.

**Table 1:** Summary of the causes and imaging findings of vascular TTS.

Vascular cause	Description	Key ultrasound findings
Plantar varicose veins	Dilated, tortuous plantar veins (> 5 mm diameter) associated with venous stasis	Dilated vein in direct contact with the tibial or plantar nerve; increased diameter when standing; Doppler showing slow flow or venous reflux
Tibial venous aneurysm	Focal dilatation of the tibial vein in direct contact with the tibial nerve	Fusiform or saccular anechoic area within the vein; turbulent flow on Doppler; clear compression of the adjacent nerve
Venous thrombosis	Intraluminal thrombus leading to venous dilatation and nerve compression	Non-collapsible vein with echogenic material; absence of intraluminal flow on Doppler; close anatomical relationship with the nerve
Tibial artery kinking	Tortuous or kinked tibial artery impinging on the nerve	Tortuous arterial course; pulsatile signal; direct contact with the tibial nerve; normal or altered flow direction on Doppler
Vascular malformation	Venous malformations (e.g., Klippel-Trénaunay syndrome) compressing the nerve within the tarsal tunnel	Dilated veins with anomalous channels; multiple venous pathways; direct contact with the nerve; enlargement when standing

### Differential diagnosis

Several conditions can mimic the symptoms of TTS, leading to misdiagnosis if not carefully distinguished. Among these, plantar fasciitis, Baxter’s neuropathy (inferior calcaneal nerve entrapment), and peripheral polyneuropathy are the most common. Plantar fasciitis typically presents with localized heel pain without sensory deficits, whereas Baxter’s neuropathy involves entrapment of a different nerve branch and often causes distal burning pain or numbness. In contrast, peripheral neuropathy is usually bilateral and non-focal, related to systemic causes such as diabetes. A clear understanding of the clinical features, supported by targeted ultrasound and MRI findings, is essential to accurately differentiate vascular TTS from these conditions. Table 2 summarizes these key aspects to guide diagnostic reasoning.

**Table 2:** Comparative summary of key differential diagnoses of vascular TTS, including clinical features, imaging findings, and main distinguishing aspects

Condition	Clinical features	Imaging findings	Key distinctions from vascular TTS
Plantar fasciitis	Localized heel pain, worse during first steps in the morning; no distal paresthesia	Ultrasound: thickened, hypoechoic plantar fascia; normal tibial nerve MRI: increased signal at insertion site	No nerve compression; symptoms localized to plantar fascia
Baxter's neuropathy	Burning heel pain, medial heel numbness, sometimes radiating to the lateral foot	Ultrasound: thickening or swelling of inferior calcaneal nerve; possible entrapment beneath abductor hallucis MRI: may show atrophy of abductor digiti minimi	More distal entrapment; affects inferior calcaneal nerve, not tibial nerve directly
Peripheral neuropathy	Bilateral, diffuse sensory loss; associated with systemic conditions (e.g., diabetes)	Ultrasound: no focal compressive site; possible diffuse nerve enlargement MRI: diffuse signal changes	Non-focal; lacks dynamic vascular compressive factor; often bilateral

### Pitfalls

**Figure 6:**
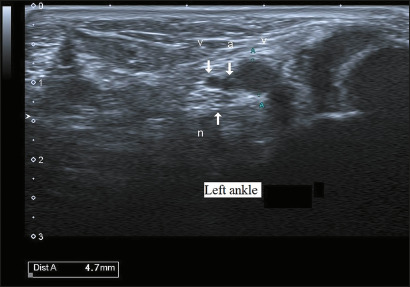
Pitfall in vascular TTS diagnosis in the left ankle. Ultrasound image demonstrating a mildly dilated tibial vein adjacent to the tibial nerve, without signs of nerve compression or displacement. This presentation may result in false-positive diagnoses if not carefully correlated with clinical findings and the dynamic assessment. (v, vein; a, artery; n, nerve).

Pitfalls and false positives are important considerations in the ultrasonographic diagnosis of vascular TTS. Dilated veins within the tarsal tunnel are not uncommon and may be seen in asymptomatic patients, particularly those with chronic venous insufficiency (Figure 6). For example, mild varicose plantar veins that do not directly impinge on the tibial nerve may appear significant on imaging but do not correlate with symptoms. Similarly, positional changes during scanning can transiently enlarge veins without true nerve compression. Inexperienced operators may also misinterpret normal vascular variants, such as prominent medial plantar veins, as pathological findings. Therefore, careful clinical correlation, dynamic scanning in the supine and standing positions, and recognition of nerve contact or displacement are essential to avoid overdiagnosis and unnecessary interventions.

### Limitations

Ultrasonography is highly effective but has limitations. It may not detect muscle edema due to denervation or evaluate the severity and functional impact of neuropathy. For soft tissue masses or detailed assessments, MRI with gadolinium contrast continues to be the preferred modality. The diagnostic utility of ultrasonography may also be reduced in cases of significant obesity. In addition, muscle atrophy and fatty infiltration are harder to diagnose on ultrasound than on MRI, requiring comparison with the contralateral foot^(4)^.

### Management and treatment

Nonsurgical treatments, including activity modification, anti-inflammatory medications, orthotic inserts, and physical therapy with targeted stretching exercises, often provide symptom relief. Surgical intervention may be required when conservative methods fail, with the approach being tailored to the specific etiology of the nerve compression^(8)^.

## CONCLUSION

Vascular diseases constitute the primary cause of TTS, a condition that remains relatively unknown to most of the medical community. Therefore, although it is quite common, many patients go undiagnosed for extended periods, resulting in delayed initiation of treatment. When caused by vascular disorders, TTS is a complex condition that often poses diagnostic challenges due to its variable presentations and overlapping features with other pathologies. Varicose plantar veins, tibial venous aneurysms, thrombosis, and arterial kinking are notable vascular causes that directly or indirectly impinge on the tibial nerve, leading to neuropathic symptoms. The role of high-resolution ultrasonography is pivotal in evaluating TTS because it can demonstrate vascular and nonvascular causes of nerve compression. Early diagnosis and intervention are crucial to preventing progression and optimizing outcomes for patients with TTS caused by vascular disorders. By presenting key imaging findings and practical tips, this work has didactic value for radiologists and clinicians involved in the diagnosis of compressive neuropathies.

## Data Availability

Not applicable.
